# *SlNCED1* and *SlCYP707A2*: key genes involved in ABA metabolism during tomato fruit ripening

**DOI:** 10.1093/jxb/eru288

**Published:** 2014-07-19

**Authors:** Kai Ji, Wenbin Kai, Bo Zhao, Yufei Sun, Bing Yuan, Shengjie Dai, Qian Li, Pei Chen, Ya Wang, Yuelin Pei, Hongqing Wang, Yangdong Guo, Ping Leng

**Affiliations:** ^1^College of Agronomy and Biotechnology, China Agricultural University, Beijing 100193, PR China; ^2^Department of Chemistry and Biochemistry, University of Arizona, 1306 East University BouleVard, Tucson, USA

**Keywords:** Abscisic acid (ABA), *SlNCED1*, *SlCYP707A2*, tomato fruit ripening, tobacco rattle virus, virus-induced gene silencing (VIGS).

## Abstract

Abscisic acid (ABA) plays an important role in fruit development and ripening. Here, three *NCED* genes encoding 9-*cis*-epoxycarotenoid dioxygenase (NCED, a key enzyme in the ABA biosynthetic pathway) and three *CYP707A* genes encoding ABA 8′-hydroxylase (a key enzyme in the oxidative catabolism of ABA) were identified in tomato fruit by tobacco rattle virus-induced gene silencing (VIGS). Quantitative real-time PCR showed that VIGS-treated tomato fruits had significant reductions in target gene transcripts. In *SlNCED1*-RNAi-treated fruits, ripening slowed down, and the entire fruit turned to orange instead of red as in the control. In comparison, the downregulation of *SlCYP707A2* expression in *SlCYP707A2*-silenced fruit could promote ripening; for example, colouring was quicker than in the control. Silencing *SlNCED2/3* or *SlCYP707A1/3* made no significant difference to fruit ripening comparing RNAi-treated fruits with control fruits. ABA accumulation and *SlNCED1*transcript levels in the *SlNCED1*-RNAi-treated fruit were downregulated to 21% and 19% of those in control fruit, respectively, but upregulated in *SlCYP707A2*-RNAi-treated fruit. Silencing *SlNCED1* or *SlCYP707A2* by VIGS significantly altered the transcripts of a set of both ABA-responsive and ripening-related genes, including ABA-signalling genes (*PYL1, PP2C1*, and *SnRK2.2)*, lycopene-synthesis genes (*SlBcyc*, *SlPSY1* and *SlPDS*), and cell wall-degrading genes (*SlPG1, SlEXP*, and *SlXET*) during ripening. These data indicate that *SlNCED1* and *SlCYP707A2* are key genes in the regulation of ABA synthesis and catabolism, and are involved in fruit ripening as positive and negative regulators, respectively.

## Highlight text

*SlNCED1* and *SlCYP707A2* are key genes in the regulation of ABA synthesis and catabolism, and are involved in fruit ripening as positive and negative regulators, respectively.

## Introduction

Abscisic acid (ABA) plays an important role in plant growth, stomatal movement, seed dormancy, and germination (Melcher *et al.*, 2009; Nishimura *et al.*, 2009). Moreover, it mediates adaptive responses to abiotic and biotic stresses (Chernys and Zeevaart, 2000; Shang *et al.*, 2010). At present, major progress has been made in research into the role of ABA in the regulation of fleshy fruit ripening (Rodrigo *et al.*, 2006; Zhang *et al.*, 2009a, b; Giribaldi *et al.*, 2010). These physiological processes controlled by ABA are primarily regulated by the bioactive ABA pool size, which is thought to be maintained not only by its biosynthesis, but also by its catabolism (Sawada *et al.*, 2008). The ABA metabolic pathway has been established by genetic approaches. ABA is synthesized *de novo* from a C_40_ carotenoid. The carotenoid-biosynthetic pathway begins with the formation of phytoene from two molecules of geranylgeranyl diphosphate (GGPP) in the central isoprenoid pathway. Four desaturation steps give rise to lycopene; cyclizations at both ends of the lycopene molecule produce α-or β-carotene, which undergo hydroxylation at C3 and C3′ to form the xanthophylls lutein and zeaxanthin, respectively. An important phase of ABA biosynthesis is initiated in plastids with the hydroxylation and epoxidation of the β-carotene to produce the all-*trans* xanthophylls zeaxanthin and violaxanthin. Violaxanthin is then converted into 9-*cis*-epoxyxanthophylls, which are oxidatively cleaved by 9-*cis*-epoxycarotenoid dioxygenase (NCED) to yield xanthoxin, the first C_15_ intermediate of ABA biosynthesis. Xanthoxin exits the plastid into the cytosol where it is oxidized in two further steps to form ABA (Schwartz *et al.*, 2003; Taylor *et al.*, 2005). In addition, the ABA metabolic pathway plays an important role in the regulation of ABA levels (Cutler and Krochko, 1999; Schwartz *et al.*, 2003). The hydroxylation pathway is the main ABA catabolic pathway. In the hydroxylation pathway, among three different methyl groups, C8′ is the predominant position for the hydroxylation reaction, which is mediated in *Arabidopsis* by proteins encoded by the *CYP707A* gene family (Saito *et al.*, 2004; Kushiro *et al.*, 2004). Dihydroxyphaseic acid (DPA) may be the major metabolite of ABA (Setha *et al.*, 2005). The 8-hydroxylation is reported to be the major regulatory step in many physiological events controlled by ABA (Kushiro *et al.*, 2004; Saito *et al.*, 2004). Recently, a major breakthrough in the field of ABA signalling was achieved with the identification of the PYR/PYL/RCAR protein family, the type 2C protein phosphatases (*PP2Cs*), and subfamily 2 of the SNF1-related kinases (*SnRK2*s) (Ma *et al.*, 2009; Park *et al.*, 2009; Melcher *et al.*, 2009; Santiago *et al.*, 2009). To elucidate the mechanism of ABA action, it is necessary to identify all the components involved in ABA homeostasis, including the functional components in ABA metabolic pathways, signal transduction, and transport.

In tomato (*Solanum lycopersicum*), three *NCED* genes have been isolated, and expression analysis indicated that, among them, *SlNCED1* may regulate ABA biosynthesis in fruit (Sun *et al*., 2012a, b). However, the molecular evidence remains to be elucidated. To address this, a key step in ABA biosynthesis, NCED was targeted for inhibition via RNAi in tomato fruit two years previously. Four independent transgenic plants were evaluated (Sun *et al*., 2012a, b). Our data showed that ABA potentially regulated the development and ripening of tomato fruit. In addition, ABA could control, at least in part, the production and effects of ethylene in climacteric tomato fruit. In recent years, tobacco rattle virus-induced gene silencing (VIGS) has been used as a rapid gene function assay system in molecular biological studies of ﬂeshy fruit (Fu *et al.*, 2006; Hoffmann *et al.*, 2006; Li *et al.*, 2013). In this study, we further identify the function of three *NCED* and three *CYP707A* genes by VIGS. Results show that *SlNCED1* and *SlCYP707A2* are key genes in the regulation of ABA level during development and ripening of tomato fruit.

## Materials and methods

### Construction of the viral vector and agroinoculation

The pTRV1 and pTRV2 virus-induced gene silencing vectors (described by Liu *et al.*, 2002) were kindly provided by YL Liu (School of Life Science, Tsinghua University, Beijing, China). A 456bp cDNA fragment of an *NCED* or *CYP707A* gene was ampliﬁed using primers (Table 1). The ampliﬁed fragment was cloned into EcoRI/SacI-digested pTRV2. *Agrobacterium tumefaciens* strain GV3101 containing pTRV1, pTRV2, and the pTRV2-derivative pTRV2-*NCED/CYP707A* were used for RNAi. Thirty fruits from ten independent plants grown in the greenhouse were selected for inoculation, and each basal pedicel or one side of fruit was injected with the *NCED/CYP707A*-RNAi TRV vector at the maturation green stage. The fruits were evaluated 3–12 days after treatment.

**Table 1.** Specific primers used for VIGS in this study

Target gene	Oligonucleotides (5′-3′)
SlNCED1 F-v	CGGGAATTCTAGTTACGCTTGCCGTTTCACTGAA
SlNCED1 R-v	CGGGAGCTCTCAGGAATGACGACGAAGTTCTCAG
SlNCED2 F-v	CGGGAATTCACAAGACGACAACTACTTTCACCCT
SlNCED2 R-v	CGGGAGCTCTCTAGAGTCATGGCATTTACAATTTG
SlNCED3 F-v	CGGGAATTCTCCACGACCCGAATAAAGTATCT
SlNCED3 R-v	CGGGAGCTCGTCTTGTTTACTTGTCCCGCTTC
SlCYP707A1 F-v	CGGGAATTCCATTTGGATGGTCATGTTAAGGA
SlCYP707A1 R-v	CGGGAGCTCCAAATAACTTTCTGTTCAGCCTTGA
SlCYP707A2 F-v	CGGGAATTCTCTTTGCAGCTCGAGACACTACTGC
SlCYP707A2 R-v	CGGGAGCTCTCCAGCTTGGCTAAGTCATTCCCT
SlCYP707A3 F-v	CGGGAATTCTTTCAAGACTCACATATTGGGATG
SlCYP707A3 R-v	CGGGAGCTCTAGCACCTCTTTAGATCCTCCCT

### Plant materials

Tomatoes (*Solanum lycopersicum* L. cv. JiaBao) were grown under standard greenhouse conditions (25±5°C and 70% humidity under a 14h/10h light/dark regime). Fruit ripening stages were divided according to the days after flowering (DAF) and fruit colour: immature green (IM), 15 DAF; mature green (MG), 30 DAF; breaker 0 (B0), 34 DAF; breaker 1 (B1), 35 DAF; breaker 2 (B2), 36 DAF; turning 0 (T0), 37 DAF; turning 3 (T3), 40 DAF; red ripe (R), 42 DAF; and over ripe (OR), 45 DAF. Ten fruits were harvested at each stage and immediately frozen in liquid nitrogen. They were then powdered, mixed, and stored at –80^o^C until further use. ‘Tom’ tomatoes were also grown in the same greenhouse.

### Dehydration treatment of fruits

In order to evaluate the effect of dehydration stress, 60 fruits were harvested at the MG stage and divided evenly into two groups. The first group (control) was stored at 20°C under high relative humidity (RH) (95%) which included 10 control fruits, 10 *SlNCED1*-RNAi-treated fruits, and 10 *SlCYP707A2*-RNAi-treated fruits. The second group (dehydration stress) was stored at the same temperature and subjected to the same treatments, but under low RH (55%, dehydrated fruits). The ABA content and expression of related genes in the pulp were determined 0, 3, 5, and 7 d after the treatment of fruits and 0, 1, and 2 d after the treatment of sepals. Every single fruit was weighed immediately after harvest and then weighed again before sampling for calculation of water loss rate. Water loss rate is calculated as the ratio of the decreased fruit weight to the initial fruit weight.

### ABA or NDGA treatment

Sixty fruits harvested at the MG stage were divided into two groups (*n* = 30 for each group), and immediately soaked in 100 µM ABA (Sigma, A1049, USA) (group I) or distilled water (group II, control) for 10min under low vacuum. The fruits were then placed in a tissue culture room at 25°C and 95% RH. After 0, 1, and 3d, the fruits were sampled, frozen with liquid nitrogen, powdered, mixed, and stored at –80^o^C for further use. Different treatments with nordihydroguaiaretic acid (NDGA) were the same as with ABA; NDGA concentration was 200 µM.

### Quantitative real-time PCR analysis

Total RNA was isolated from tomato samples using the hot borate method (Wan and Wilkins, 1994). Genomic DNA was eliminated using an RNase-free DNase I kit (Takara, China) according to the manufacturer’s recommendations. For every RNA sample, quality and quantity were assessed by agarose gel electrophoresis. cDNA was synthesized from total RNA using the PrimeScript^TM^ RT reagent kit (Takara) according to the manufacturer’s recommendations. Primers used for real-time PCR are listed in Supplementary Table S1 and were designed using Primer 5 software (http://www.premierbiosoft.com/). *SAND* was used as an internal control gene, and the stability of its expression was tested in preliminary studies (Sun *et al.*, 2011). All primer pairs were tested by PCR. The presence of a single product of the correct size for each gene was conﬁrmed by agarose gel electrophoresis and double-strand sequencing (Invitrogen). The ampliﬁed fragment of each gene was subcloned into the pMD18–T vector (Takara), and used to generate standard curves through serial dilution. The real-time PCR was performed using a Rotor-Gene 3000 system (Corbett Research, China) with SYBR Premix Ex Taq^TM^ (Takara). Each 20 µl reaction solution contained 0.8 µl of primer mixer (containing 4 µM of each forward and reverse primer), 1.5 µl cDNA template, 10 µl SYBR Premix Ex Taq^TM^ (2X) mixer, and 7.7 µl water. Reactions were performed under the following conditions: 95°C for 30 s (one cycle), 95°C for 15 s, 60°C for 20 s, and 72°C for 15 s (40 cycles). The changes of relative fold expression were calculated using the relative two standard curves method with Rotor-Gene 6.1.81 software (Invitrogen).

### Determination of ABA content

For ABA extraction, 1.0g of pulp was ground in a mortar and homogenized in the extraction solution (80% v/v methanol). Extracts were centrifuged at 10 000*g* for 20min. The supernatant was eluted through a Sep-Pak C_18_ cartridge (Waters, www.waters.com) to remove polar compounds, and then were stored at 20°C for ELISA. The stepwise procedure for indirect ELISA of ABA was as follows: each well of a microtitre plate was pre-coated with ABA–BSA conjugater diluted in coating buffer according to the instructions of the manufacturer (ELISA kit for ABA, College of Agronomy and Biotechnology, China Agricultural University). Then, to each well, was added 50 µl standard or sample in assay buffer (8.0g NaCl, 0.2g KH_2_PO_4_, 2.96g Na_2_HPO_4_12 H_2_O, 1.0ml Tween 20, and 1.0g gelatin, added to 1.000ml water), followed by 50 µl ABA antibody (Invitrogen) diluted 1:2000 in assay buffer. The plates were incubated for 0.5h at 37°C and then washed four times with scrubbing buffer (which contained the same ingredients as assay buffer, but without gelatin). Anti- mouse IgG coupled to alkaline phosphatase (100ml of a 1:1000 dilution) was added to each well, and the plates were incubated for 0.5h at 37°C. The plates were washed as above, and then 100 µl of a 1–2mg ml^–1^ o-phenylenediamine substrate solution and 0.04% by volume of 30% v/v hydrogen peroxide in substrate buffer (5.10g C_6_H_8_O_7_H_2_O, 18.43g Na_2_HPO_4_12 H_2_O, and 1.0ml Tween 20, added to 1000ml water) were added to each well. After 10–15min, 50 µl of 2.0mol l^–1^ H_2_SO_4_ was added to each well to terminate the reaction. The absorbance was measured at 490nm using a Thermo Electron (Labsystems) Multiskan MK_3_ (Pioneer, www.pioneerbiomed.com). The concentration of ABA in the sample was calculated from log B/B_0_-transformed standard curve data, where B and B_0_ are the absorbance values with or without the competing antigen, respectively.

### Determination of ethylene production

The ethylene production of the fruit was measured by enclosing three fruits in 1.0 l airtight containers for 2h at 20°C, withdrawing 1ml of the headspace gas, and injecting it into a gas chromatograph (Agilent model 6890N) ﬁtted with a ﬂame ionization detector and an activated alumina column. Fresh tissues from each fruit were frozen in liquid nitrogen and stored at –80°C until use.

### Determination of fruit firmness

Fruits were harvested from all of the plants in each of three replicate plantings at the different ripening stages. Flesh firmness was measured after the removal of fruit skin on three sides of each fruit using a KM-model fruit hardness tester (Fujihara). The strength of flesh firmness was recorded in kg cm^–1^. Compression of each fruit was measured three times, and the average of the maximum force was used.

## Results

### Expression patterns of ABA metabolic genes in pulp during fruit development and in response to application of exogenous ABA and dehydration stress

**Fig. 1.**
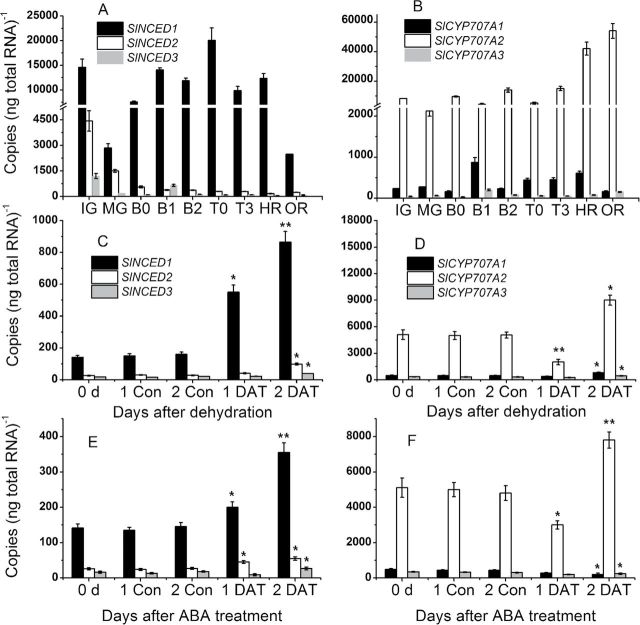
Changes in the expression of *SlNCEDs* and *SlCYP707As* during fruit development, and in response to dehydration stress and the application of exogenous ABA. (A, B) Absolute transcript level profiles for both *SlNCED*s and *SlCYP707A*s during fruit development. IG, 15 days after anthesis (DAA); MG, 30 DAA; B0 to B2, 31 to 35 DAA; T0 and T3, 36 to 37 DAA; HR, 38 DAA; and OR, 40 DAA. (C, D) Effect of dehydration stress on the expression of *SlNCED*s and *SlCYP707A*s. Fruits were harvested at the MG stage, divided into two groups, and then placed in an incubator at 25^o^C. The relative humidity for group 1 is 90% as the control, and 45% for group 2 as the dehydration treatment. Each treatment lasted 4 d and the samples were collected at 1 and 2 d after treatment (DAT). (E, F) Effect of exogenous ABA treatment on the expression of *SlNCED*s and *SlCYP707A*s. Fruits harvested at the MG stage were divided into two groups and treated with ABA (100mM) or distilled water for 10min. The samples were collected at 1 and 2 DAT. *SAND* mRNA was used as the internal control. Three biological replicates (*n* = 3) were used for each analysis. *P value *t*-test < 0.05; **P value *t*-test < 0.001. Error bars are SE.

Within the *SlNCED* gene family, the expression of *SlNCED1* was the highest in pulp. *SlNCED1* decreases from 10 days after full bloom (DAFB) to the MG stage, then it increased sharply and peaked at the turning stage; after that it declined to a low level at the OR stage (Fig. 1A). The expression variation of *SlNCED2* was generally decreased from 10 DAFB to fruit ripening. The expression of *SlNCED3* was very low through fruit development and ripening. Among the *SlCYP707A* gene family, the expression of *SlCYP707A2* (Fig. 1B) exhibited a fluctuant expression pattern with four peaks during development, and then it increased rapidly during ripening. Compared to *SlCYP707A2*, the expression of *SlCYP707A1* and *SlCYP707A3* (Fig. 1B) was very low during fruit development and ripening. In addition, expression of ABA metabolic genes in response to exogenous ABA treatment and dehydration in tomato fruits was tested. For *SlNCED1*, expression was significantly increased by exogenous ABA and dehydration at 2 days after treatment (DAT) (Fig. 1C, E). The expression of *SlNCED2* and *SlNCED3* was also significantly increased in the water stressed and ABA-treated tomato fruits (Fig. 1C, E). For *SlCYP707A2*, expression was downregulated in the fruits under both ABA treatment and dehydration at 1 DAT (Fig. 1D, F), and then it significantly increased at 2 DAT (Fig. 1D, F). The expression of *SlCYP707A1* and *SlCYP707A3* was significantly downregulated under both water stress and ABA treatment at 1 DAT, but upregulated at 2 DAT.

### Silencing of the *SlNCED1* gene suppresses tomato fruit ripening

**Fig. 2.**
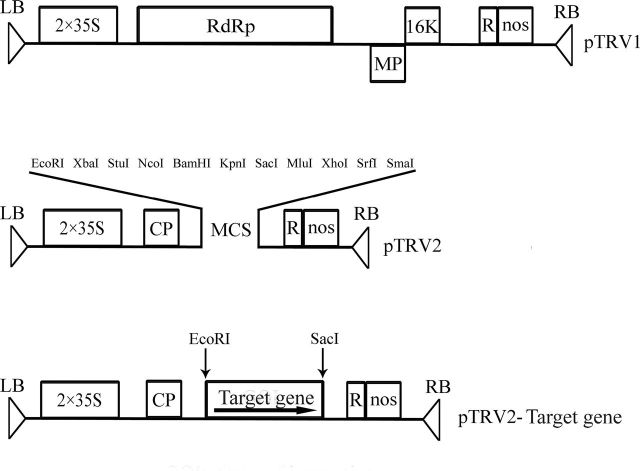
Construction of pTRV1, pTRV2, and pTRV2-derivative pTRV2-target gene. The TRV-based virus-induced gene silencing vectors are as described by Liu *et al.* (2002). TRV cDNA clones were placed between duplicated CaMV 35S promoters and the nopaline synthase terminator in a T-DNA vector. The pTRV2-target gene (sense orientation) was constructed to assess the ability of TRV vectors to suppress expression of the target gene in tomato fruits. RdRp, RNA-dependent RNA polymerase; 16K, 16kDa cysteine-rich protein; MP, movement protein; CP, coat protein; LB and RB, left and right borders of T-DNA, respectively; R, self-cleaving ribozyme; MCS, multiple cloning sites.

**Fig. 3.**
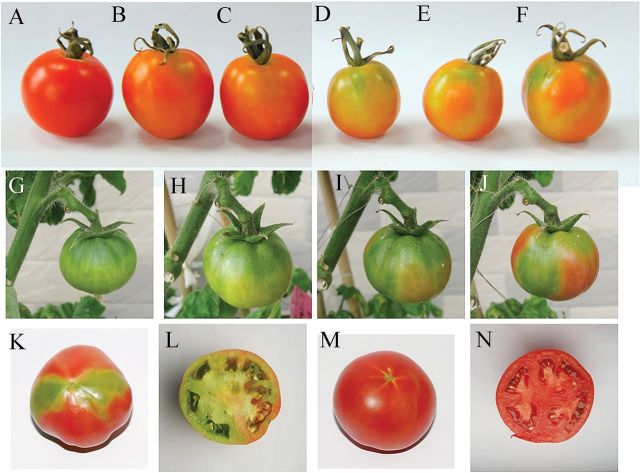
Phenotype of *SlNCED1/2/3-RNAi*-treated fruits. At the maturation green stage, 48 tomato fruits (cv. Tom) from 10 independent plants grown in the greenhouse were selected and divided into four groups (each group has 12 fruits for three repetitions; each repetition has four fruits) for VIGS testing. The *SlNCED1*-RNAi, *SlNCED2*-RNAi, and *SlNCED3* -RNAi TRV vectors were injected from the pedicel into group 1, group 2, and group 3, respectively. Group 4 was injected with TRV vector (no *NCED* gene) as a control. The fruits were evaluated 3–12 d after inoculation. Panels A to F show Tom tomato fruits 9 d after VIGS treatments: (A) Control; (B) *SlNCED2*-RNAi-treated fruit; (C) *SlNCED3*-RNAi-treated fruit; (D–F) *SlNCED1*-RNAi-treated fruit. Panels G–N show JiaBao tomato fruits treated with *SlNCED1*-RNAi: (G) MG stage fruit (before treatment); (H) 5 d days after inoculation (DAI); (I) 7 DAI; (J) 9 DAI; (K, L) 12 DAI; (M, N) 12 days after MG stage, control fruit.

To examine the role of *SlNCEDs*, the tobacco rattle virus (TRV) vector was used to suppress the expression of *SlNCED*s (Fig. 2). When the *SlNCED1*-RNAi TRV vector was injected into the basal pedicel of 15 fruits (cv. Tom) attached to the plant at the MG stage, fruit ripening slowed down, and the entire fruit turned orange (Fig. 3D, E,F) instead of red as in the control (Fig. 3A). Ten days after the *SlNCED1*-RNAi TRV vector was injected, the fruits did not show normal ripening (Fig.3D, E, F) as in the control (Fig. 3A). Compared to *SlNCED1*-RNAi-treated fruits, there were no significant differences in colouring between *SlNCED2/3*-RNAi-treated fruits and control fruits (Fig. 3B, C) during fruit ripening and in response to dehydration stress. The *SlNCED1*-RNAi TRV vector was also injected into the attached fruits (cv. JiaBao) at the MG stage. 12 d after injection, control fruits turned red (Fig. 3M, N); however, for *SlNCED1*-RNAi-treated fruits, parts of the peel and placenta inside the fruit did not turn red as in the control (Fig.3K, L). The water loss of the sepal in the *SlNCED1*-RNAi-treated fruits was quicker than in the control under the same conditions. Meanwhile, the wilting of the sepals was more serious than that of the control under the same conditions.

### SlNCED1-*RNAi treatment alters the expression of genes involved in ABA-responsive genes*

**Fig. 4.**
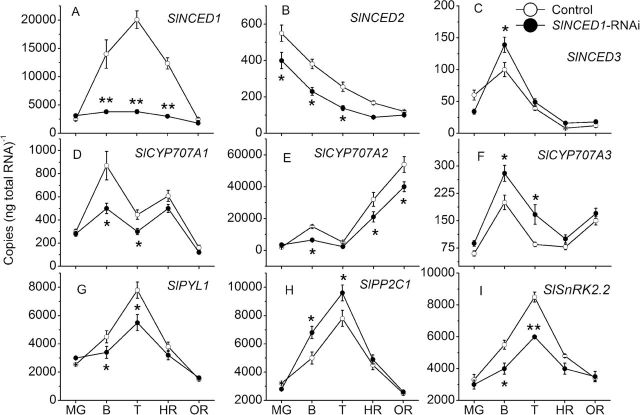
Expression of ABA-responsive genes in both control and *SlNCED1-*RNAi-treated fruit during development and ripening of tomato. The JiaBao fruits were injected with *SlNCED1*-RNAi TRV vectors at THE MG stage. Fruits were sampled 0 d (MG), 5 d (B), 7 d (T), 9 d (HR), and 12 days (OR) after the inoculation, respectively. *SAND* mRNA was used as the internal control. Three biological replicates (*n* = 3) were used for each analysis. *P value *t*-test < 0.05; **P value *t*-test < 0.001. Error bars are SE.

**Fig. 5.**
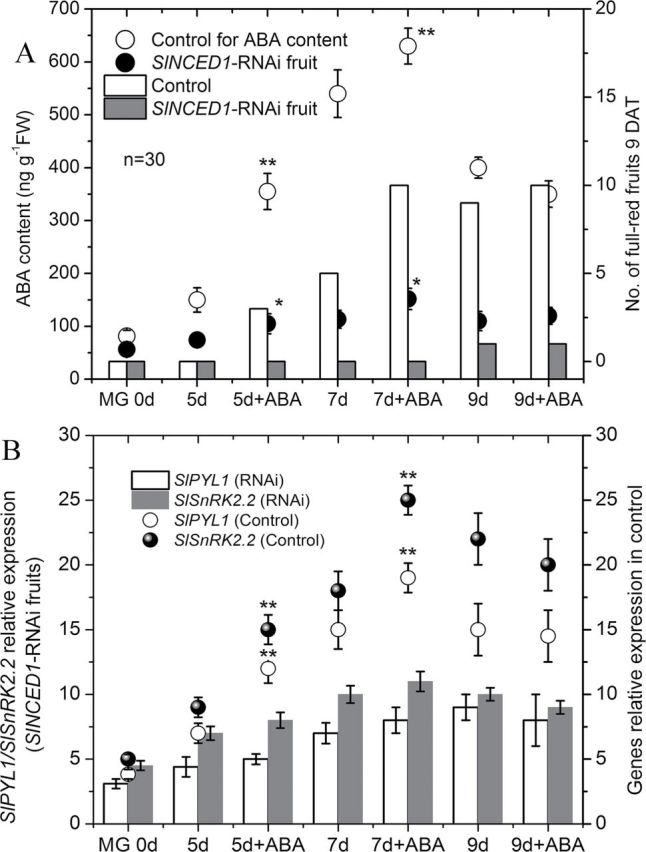
Changes in ABA content, *SlPYL1* and *SlSnRK2.2* expression, and numbers of fully red fruit 9 d after exogenous ABA treatment in both control and *SlNCED1-*RNAi-treated fruit. 78 JiaBao fruits were equally divided into two groups at the MG stage and then injected with 1ml ABA (100mM) for group 1, or distilled water for group 2 (control). 30 fruits were used for the investigation of number of fully red fruits and others were sampled at 0, 5, 7, and 9 d after ABA treatment, respectively, for the determination of ABA content and gene expression. *SAND* mRNA was used as the internal control. Three biological replicates (*n* = 3) were used for each analysis. *P value *t*-test < 0.05; **P value *t*-test < 0.001. Error bars are SE.

In *SlNCED1*-RNAi-treated fruits, expression of *SlNCED1* was markedly downregulated to 19% of the control while expression of *SlNCED2*/*SlNCED3* was downregulated/upregulated (Fig. 4A, B, C). In fruits with the same treatment, the expression of *SlCYP707A1/2* was downregulated, while the expression of *SlCYP707A3* was upregulated (Fig.4D, E, F). Among the ABA-signalling genes, including those of the PYR/PYL/RCAR protein family (*SlPYL1*), type 2C protein phosphatases (*SlPP2C1*), and subfamily 2 of SNF1-related kinases (*SlSnRK2.2*) (Ma *et al.*, 2009; Park *et al.*, 2009), *SlPYL1*and *SlSnRK2.2* were downregulated while *SlPP2C1* was upregulated (Fig. 4G, H, I). As shown in Fig. 5A, in *SlNCED1*-RNAi-treated fruits, the ABA content was 21% of the control at the turning stage (7 d after MG); moreover, fruits couldn’t become fully red and this incomplete colouring couldn’t be rescued by the application of exogenous ABA (although ABA contents were increased by application of exogenous ABA) (Fig. 5A). Expression of *SlPYL1* and *SlSnRK2.2* in *SlNCED1*-RNAi-treated fruits couldn’t be increased by application of exogenous ABA (Fig. 5B).

### SlNCED1-*RNAi treatment alters the expression of genes involved in ripening-related genes*

**Fig. 6.**
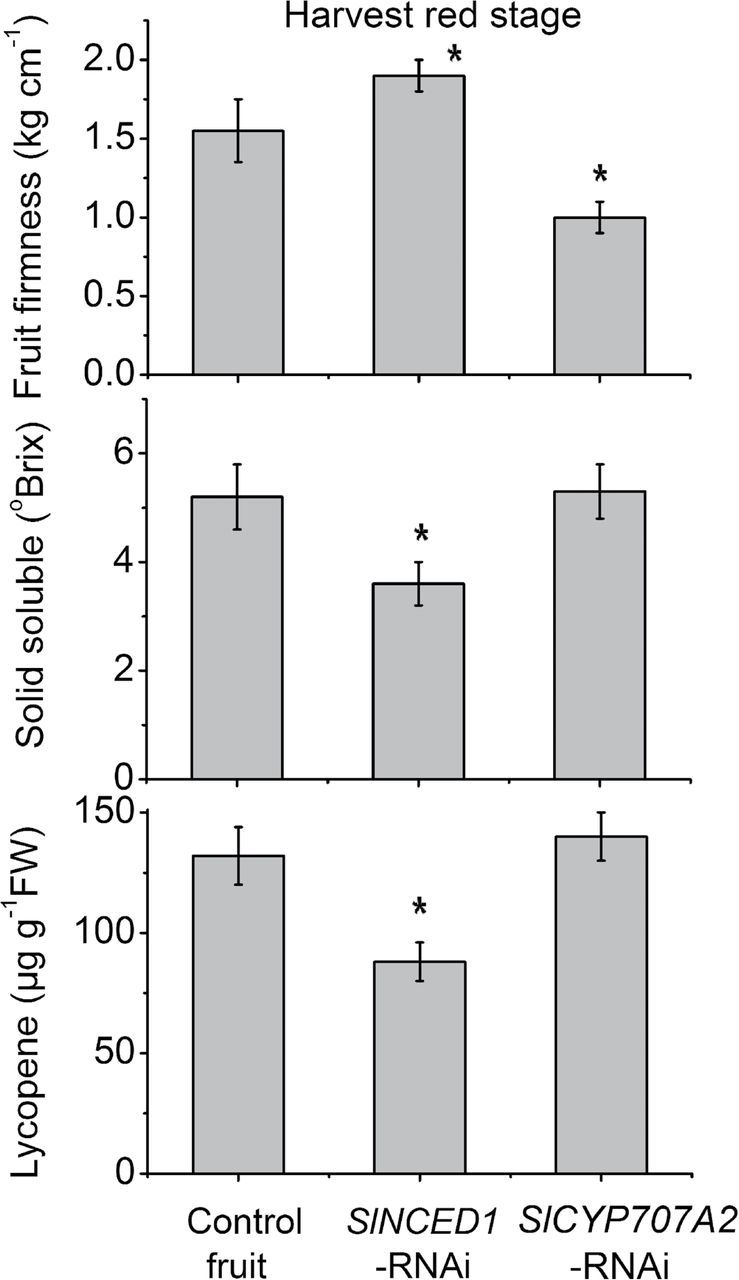
Changes in fruit firmness, solid soluble, and lycopene content in control, *SlNCED1-*RNAi-treated, and *SlCYP707A2-*RNAi-treated fruits. Every nine JiaBao fruits from control, *SlNCED1-RNAi*-treated, and *SlCYP707A2-*RNAi-treated fruits, respectively, were harvested at the harvest red stage. Three biological replicates (*n* = 3) were used for each analysis. *P value *t*-test < 0.05; **P value *t*-test < 0.001. Error bars are SE.

**Fig. 7.**
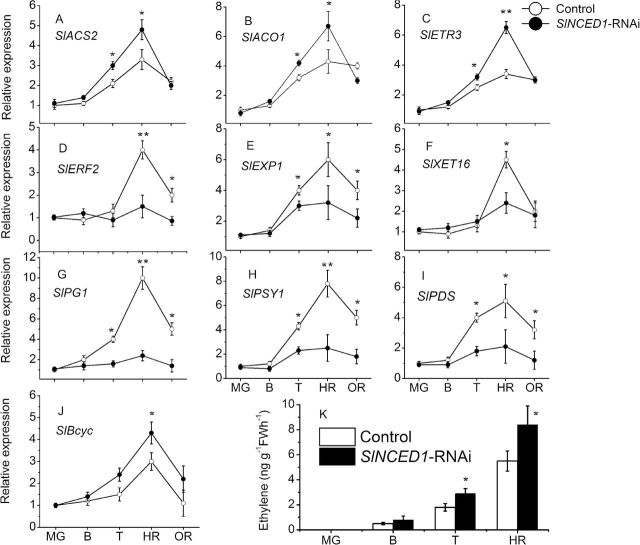
Changes in ethylene release and relative expression of genes involved for ethylene, cell wall catabolism, and lycopene synthesis in both control and *SlNCED1-RNAi*-treated fruits during development of tomato. The JiaBao fruits were injected with *SlNCED1*-RNAi TRV vectors at the MG stage. Fruits were sampled 0d (MG), 5d (B), 7d (T), 9d (HR), and 12 d (OR) after the inoculation, respectively. *SAND* mRNA was used as the internal control. Three biological replicates (*n* = 3) were used for each analysis. *P value *t*-test < 0.05; **P-value *t*-test < 0.001. Error bars are SE.

Several ripening-related physiological parameters were measured, including fruit firmness, solid soluble content, and lycopene content. As shown in Fig. 6, in *SlNCED1*-RNAi fruits, the trends were for a decrease in solid soluble content and lycopene content, but an increase in fruit firmness. To clarify the role of *SlNCED1* in the regulation of tomato fruit ripening, several ripening-related genes were examined in both *SlNCED1-*RNAi-treated fruits and control fruits. *SlBcyc*, *SlPSY1* and *SlPDS* encode lycopene β-cyclase, phytoene synthetase, and phytoene dehydrogenase, respectively. Relative quantitative real-time PCR analysis showed that the expression of all these genes was downregulated (Fig. 7H, I) except for *SlBcyc*, which was upregulated (Fig. 7J). In addition, we examined the expression of genes encoding cell-wall hydrolases. In *SlNCED1*-RNAi fruit, genes encoding polygalacturonase (*SlPG1*), expansin (*SlEXP1*), and xyloglucan endotransglycosylase (*SlXET16*) were all significantly downregulated during fruit ripening compared to the control (Fig. 7E, F, G). Relative expression analysis showed that the expressions of *SlACS2* [encoding 1-aminocyclopropane-1-carboxylic acid (ACC) synthase], *SlACO1* (encoding ACC oxidase), and *SlETR3* (involved in the ethylene response), were upregulated in the *SlNCED1*-RNAi fruits (Fig. 7A, B, C). The expression of *SlERF2* was significantly downregulated in *SlNCED1*-RNAi fruits (Fig. 7D). Ethylene release was upregulated at turning stage, but downregulated at harvest red stage compared to the control (Fig. 7K).

### *Silencing of the* SlCYP707A2 *gene promotes tomato fruit colouring*

**Fig. 8.**
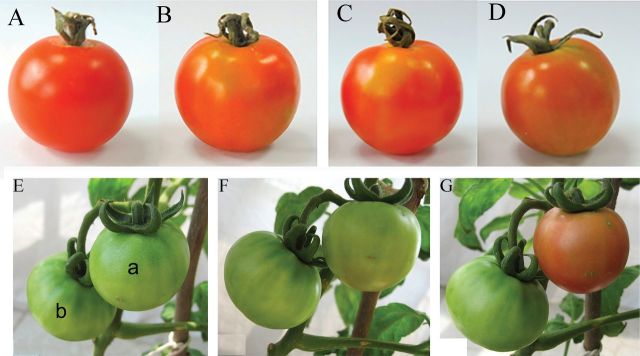
Phenotype of *SlCYP707A1/2/3-*RNAi-treated fruits. At the MG stage, 48 tomato fruits (cv. Tom) from 10 independent plants grown in the greenhouse were selected and divided into four groups (each group, 12 fruits for three repetitions; each repetition, four fruits) for the VIGS test. The *SlCYP707A1-*RNAi, *SlCYP707A2-*RNAi, and *SlCYP707A3-*RNAi TRV vectors were injected from the pedicel into group 1, group 2, and group 3, respectively. Group 4 was injected with TRV vector (no *CYP707A* gene) as a control. The fruits were evaluated 3–12 d after inoculation. Panels A–D are Tom tomato fruits. (A) Control; (B) *SlCYP707A1-*RNAi-treated fruit; (C) *SlCYP707A3-*RNAi-treated fruit; (D*) SlCYP707A2-*RNAi-treated fruit. Panels E to G are JiaBao tomato fruits. (E) 30 days after anthesis (before treatment; a, *SlCYP707A2-*RNAi fruit; b, control fruit); (F) 3 d after *SlCYP707A2-*RNAi treatment; G, 6 d after *SlCYP707A2-*RNAi treatment.

**Fig. 9.**
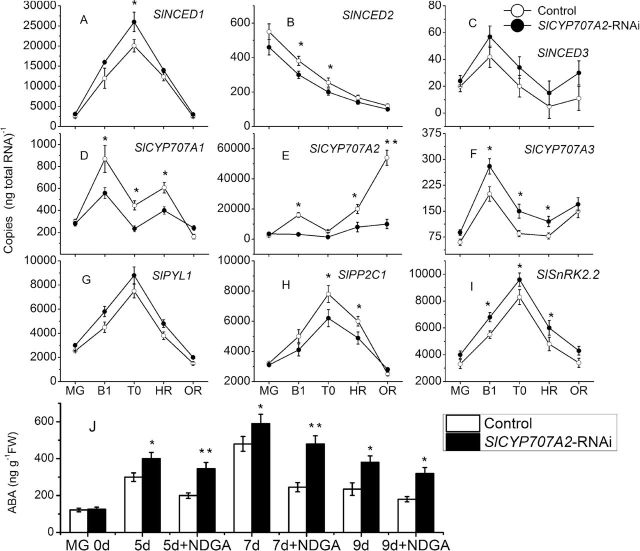
Changes in the expression of genes related to ABA metabolism and signalling in both control and *SlCYP707A2-*RNAi-treated fruits during development, and the ABA contents in fruits under the application of NDGA. For panels A to I, JiaBao fruits were injected with *SlCYP707A2*-RNAi TRV vector at the MG stage. Fruits were sampled 0 d (MG), 5 d (B), 7 d (T), 9 d (HR), and 12 d (OR) after the inoculation, respectively. (J) Fruits on plants were divided into two groups and then injected with 1ml NDGA (200mM) or distilled water at the MG stage. The samples were collected at 0, 5, 7, and 9 d after treatment. Three biological replicates (*n* = 3) were used for each analysis. *P value *t*-test < 0.05; **P value *t*-test < 0.001. Error bars are SE.

To clarify the role of *SlCYP707A2* in the regulation of ABA levels during fruit ripening, ABA levels and ABA-responsive genes were examined in both RNAi-treated and control fruits. When the *SlCYP707A2* -RNAi TRV vector was injected into the 15 fruits attached to the plant at the MG stage, the fruits could become red quicker (Fig. 8F), and ripening was also faster than in the control (Fig. 8G). In *SlCYP707A1/2/3*-RNAi-treated Tom fruits (Fig. 8B, C, D), fruit ripening (Fig. 8B, C, D) was the same as with control fruit (Fig. 8A) at harvest stage. In *SlCYP707A2*-RNAi-treated fruits, the ABA content was higher than the control at 5 and 7 d after MG, and fruit ripening couldn’t be inhibited by the application of NDGA, which delayed the control fruit ripening (Fig. 9J).

### SlCYP707A2-*RNAi treatment alters the expression of genes involved in ABA-responsive genes*

In the *SlCYP707A2*-RNAi-treated fruits, expression of *SlCYP707A1* was downregulated, but *SlCYP707A3* was upregulated; however, *SlCYP707A2* was markedly downregulated to 18% of the control (Fig. 9D, E, F). The expression of *SlNCED1/3* was upregulated, while *SlNCED2* was downregulated in *SlCYP707A2*-RNAi fruits (Fig. 9A, B, C). The expression of *SlPYL1* and *SlSnRK2.2* in ABA signalling was upregulated, while *SlPP2C1* expression was downregulated in *SlCYP707A2*-RNAi fruit (Fig. 9G, H, I).

### SlCYP707A2-*RNAi treatment alters the expression of genes involved in ripening-related genes*

**Fig. 10.**
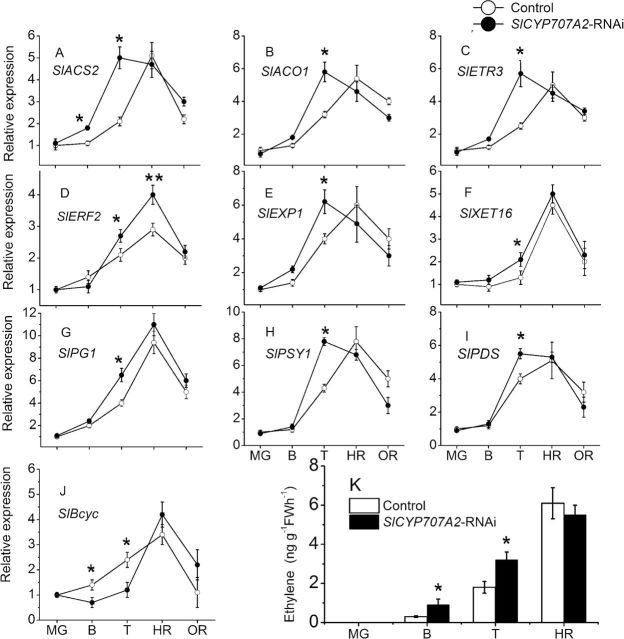
Changes in the relative expression of genes involved for ethylene, cell wall catabolism and lycopene synthesis, and ethylene release in both control and *SlCYP707A2-*RNAi-treated fruits. JiaBao fruits were injected with *SlCYP707A2*-RNAi TRV vectors at the MG stage. Fruits were sampled 0 d (MG), 5 d (B), 7 d (T), 9 d (HR), and 12 d (OR) after the inoculation, respectively. *SAND* mRNA was used as the internal control. Three biological replicates (*n* = 3) were used for each analysis. *P value *t*-test < 0.05; **P value *t*-test < 0.001. Error bars are SE.

As shown in Fig. 6, fruit firmness was lower than that of the control in *SlCYP707A2*-RNAi fruits, while the soluble solid content and lycopene content did not show significant differences compared to the control fruits. In *SlCYP707A2*-RNAi fruits, genes encoding polygalacturonase (*SlPG*), expansin (*SlEXP*) and xyloglucan endotransglycosylase (*SlXET16*) were upregulated at breaker and turning stages; however, there were no significant changes compared to the control fruits at the harvest stage (Fig. 10E, F, G). In the lycopene synthesis pathway, compared with the control, the relative expression levels of *SlPSY1* and *SlPDS* were higher, but *SlBcyc* was lower, at breaker and turning stages (Fig. 10H, I, J). With respect to ethylene, relative expression analysis showed that the expression of *SlACS2* [encoding (ACC) synthase], *SlACO1* (encoding ACC oxidase), and *SlETR3* (involved in the ethylene response), which were consistent with ethylene release (Fig. 10K) in the *SlCYP707A2*-RNAi fruits, were upregulated at breaker and turning stages; however, there was no significant difference at harvest stage compared to control fruits (Fig. 10J). The expression of the *SlERF2* was upregulated at the T and HR stages. *SlCYP707A2*-RNAi-treated fruits during the harvest stage were unusual, with uneven colouring in pulp compared to control fruits.

### *Dehydration of* SlNCED1/SlCYP707A2-*RNAi-treated fruits*

**Fig. 11.**
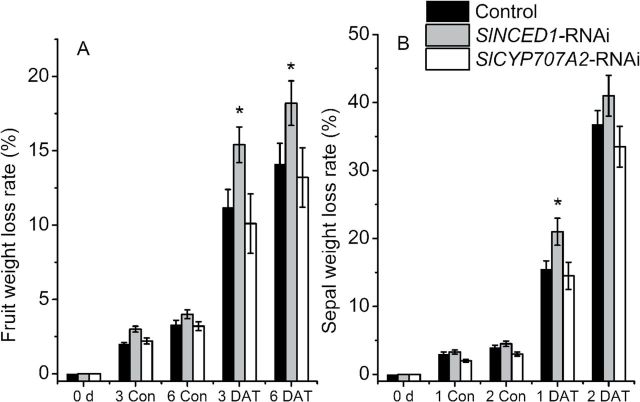
Effects of dehydration stress on control, *SlNCED1-*RNAi-treated, and *SlCYP707A2-*RNAi-treated fruits. Fruits at the MG stage were placed into an incubator: control, 25^o^C and 90% relative humidity; dehydration, 25^o^C and 45% relative humidity. Fruits were sampled 0, 3, and 6 d after dehydration treatment (DADT), respectively. The sepal samples were collected at 1 and 2 DADT. Three biological replicates (*n* = 3) were used for each analysis. *P value *t*-test < 0.05; **P value *t*-test < 0.001. Error bars are SE.

Both control and *SlNCED1*/*SlCYP707A2*-RNAi-treated fruits were harvested 6 d after the *SlNCED1*/*SlCYP707A2-*RNAi treatments (Fig. 11). The fruits were then incubated in the laboratory (20°C, 50% relative humidity). As shown in Fig. 11, the weight loss rate in *SlNCED1*-RNAi fruits was higher than the control fruit 3–6 d after dehydration. However, there was not a large difference in the rate of water loss comparing control and *SlCYP707A2*-RNAi fruit (Fig. 11A). The water loss of the sepal in the *SlNCED1*/*SlCYP707A2*-RNAi treated fruit was also similar to that of fruit under the same conditions (Fig. 11B).

## Discussion

NCED and CYP707A are generally encoded by a small gene families, respectively (Krochko *et al.*, 1998; Burbidge *et al.*, 1999; Zhang *et al.*, 2009a). Among the three *NCED* genes in tomato, *SlNCED1* may play a primary role in regulating ABA biosynthesis during fruit ripening (Fig. 1A) in response to ABA application (Fig. 1E) and dehydration (Fig. 1C). Besides biosynthesis, catabolism of ABA is also an important way of regulating ABA levels (Kushiro *et al.*, 2004; Li *et al.*, 2011; Ren *et al.*, 2011). A similar result to the reports of Ren *et al.* (2010) and Wang *et al.* (2013) was obtained in this work: the expression of *SlCYP707A2* was higher than *SlCYP707A1* and *SlCYP707A3*, and was opposite to the change of ABA content during development (Fig. 1B) in response to ABA treatment (Fig. 1F) and dehydration (Fig. 1D). Previously, to suppress *SlNCED1* specifically in tomato fruits, we used an RNA interference construct driven by the fruit-specific E8 promoter. ABA accumulation and *SlNCED1* transcript levels in transgenic fruit were downregulated to between 20 and 50% of the levels measured in control fruit. This significant reduction in NCED activity led to a downregulation in the transcription of genes encoding major cell wall-degrading enzymes, specifically polygalacturonase (SlPG), pectin methyl esterase (SlPME), and expansin (SlExp). This led to a significant extension of the shelf life to 15 to 29 d compared with a shelf life of only 7 d for the control fruit (Sun *et al.*, 2012a). Fruits of RNAi lines displayed deep red colouration compared with the pink colour of control fruit. The decrease in endogenous ABA in these transgenic lines resulted in an increase in ethylene. These results indicate that, at least in part, ABA potentially regulated fruit ripening, and ethylene production and action, in climacteric tomato fruit (Sun *et al.*, 2012b).

In the present study, we used VIGS to further verify the function of genes related to ABA metabolism in tomato fruit. The *SlNCED1*-RNAi-treated fruits showed various chimeric symptoms similar to those reported by Fu *et al.* (2006) and Hoffmann *et al.* (2006), and fruit ripening was inhibited such that fruit couldn’t become fully red or softened. Transcript levels of the *SlNCED1* gene decreased after the inoculation (RNAi), concomitant with the decrease of ABA concentration, indicating that *SlNCED1* directly affects ABA biosynthesis in tomato fruit. In addition to the *SlNCED1* gene (downregulated by 81%), most of the ABA-responsive genes were downregulated in *SlNCED1*-RNAi fruits (Fig. 4). Moreover, exogenous ABA could not rescue the uncoloured phenotype of these *SlNCED1*-RNAi-treated fruits (Fig. 5A). The ABA content was significantly upregulated in *SlNCED1*-RNAi fruit treated by exogenously applied ABA at 5 and 7 DAT (Fig. 5A). As high as the ABA content is in RNAi fruit, they still cannot complete full reddening, suggesting that may be attributed to the repressed effect on the numbers of ABA signal molecules when the *SlNCED1* is repressed by RNAi (Fig. 4). Real-time PCR analysis of the mRNA expression levels of *PYL1* and *SnRK2.2* showed that transcripts of these genes were significantly downregulated while *PP2C1* was upregulated in RNAi fruits compared with control fruits (Fig. 4). A recent report shows that in the presence of ABA, the PYR1 receptor proteins can disrupt the interaction between the SnRK2s and PP2Cs, thus preventing the PP2C-mediated dephosphorylation of SnRK2s. This results in the activation of the SnRK2 kinases, turning on downstream factors of ABA signals, such as AREB/ABF, and finally leads to ABA-responsive physiological events (Fujii *et al.*, 2009). In this study, *PYL1* and *SnRK2.2* genes were downregulated in RNAi fruit (Fig. 4) and could not be rescued by exogenous ABA treatment (Fig. 5B), which may lead to the suppression of ABA responses and, in turn, destroy ABA-responsive physiological events, such as fruit reddening and ripening.

In contrast, *CYP707A*, encoding 8′-hydroxylase, can alter the dynamic balance of ABA level (Saito *et al.*, 2004; Ren *et al.*,2011). The role of three *CYP707A* genes (*SlCYP707A1*-*3)* was verified by VIGS, and the ABA level in fruit was mainly regulated by *SlCYP707A2* at the transcriptional level (Fig. 1B). *SlCYP707A2-*RNAi caused an increase of ABA content at breaker and turning stages, and accelerated fruit colouring and softening compared to control fruits (Figs 8 and 9). This result is similar to the overexpression of *NCED* genes, which could cause the increase of ABA level (Qin and Zeevaart 2002; Wan and Li, 2006; Zhang *et al.* 2008; Hwang *et al.*, 2010). Thus, *SlCYP707A2* is a key gene in the ABA catabolic pathway in tomato fruit. *SlCYP707A1*/*2/3* can be upregulated by the application of exogenous ABA, suggesting that ABA might regulate its own accumulation in fruits (Sun *et al.*, 2012a; Wang *et al.*, 2013). Our results also suggest that appropriate ABA contents are required for fruit ripening; too much or too little could lead to abnormal ripening.

Water deficit in fruit is a direct response caused by osmotic stress, including drought and salinity. In this study, *SlNCED1-*RNAi treatment could decrease the tolerance of fruit under dehydration stress, concurrent with the decrease of ABA accumulation and increased water loss (Fig. 11). Interestingly, the effect of silencing *SlCYP707A2* in tomato fruits was different from previous findings in *Arabidopsis*. Loss of function of *CYP707A* was reported to enhance drought tolerance in *Arabidopsis* (Umezawa *et al.*, 2006). In contrast, *SlCYP707A2*-silenced tomato fruits did not show any improvements in terms of rate of weight loss of silenced fruits compared to control fruits under water stress, and this result was similar in wheat (Manmathan *et al.*, 2013). The differences in response to dehydration stress may be due to differences in stress adaptation pathways in tomato and *Arabidopsis*. Another possible reason for the observed difference may have to do with the structure of the *CYP707A* gene family in different species (Kushiro *et al.*, 2004; Manmathan *et al.*, 2013). In tomato fruits, the expression of *SlERF2*, encoding an ERF (ethylene response factor) protein and belonging to the ERF gene family, increases during fruit ripening (Fig. 7D). Individual members of the ERF family have been shown to be either positive or negative regulators of transcription (Fujimoto *et al.*, 2000; Ohta *et al.*, 2000, 2001). In *SiNCED1*-RNAi-treated fruits, *SlERF2* expression was markedly downregulated: therefore, a decrease of ethylene release at the HR stage may be influenced by *SlERF2* transcript (Fig. 7D,K).

In conclusion, six major genes including three *SlNCEDs* and three *SlCYP707As* involved in ABA biosynthesis and catabolism were identified by VIGS. VIGS-treated tomato fruits had significant reductions in target gene transcripts. The *SlNCED1*-RNAi-treated fruits did not undergo normal ripening compared to control fruits. In comparison, the *SlCYP707A2*-RNAi treatment could promote ripening; for example, colouring was quicker than the control. Silencing *SlNCED1* or *SlCYP707A2* by VIGS significantly altered the transcripts of a set of both ABA-responsive and ripening-related genes during ripening. These data indicate that *SlNCED1* and *SlCYP707A2* are key genes in the regulation of ABA synthesis and catabolism, and are involved in fruit ripening as a positive and a negative regulator, respectively.

## Supplementary material

Supplementary data can be found at *JXB* online.

Supplementary Table S1. Specific primer sequences used for real-time quantitative PCR.

## Funding

This work was partly supported by the 973 Programme 2012CB113900 to Yang-Dong Guo.

## Supplementary Material

Supplementary Data

## References

[CIT0001] Burbidge A Grieve TM Jackson A Thompson A McCarty DR Taylor IB 1999 Characterization of the ABA-deficient tomato mutant *notabilis* and its relationship with maize *Vp14.* The Plant Journal 17, 427–431. 10205899 10.1046/j.1365-313x.1999.00386.x

[CIT0002] Chernys JT Zeevaart JAD 2000 Characterization of the 9-cis-epoxycarotenoid dioxygenase gene family and the regulation of abcisic acid biosynthesis in avocado. Plant Physiology 124, 343–353. 10982448 10.1104/pp.124.1.343 PMC59148

[CIT0003] Cutler AJ Krochko JE 1999 Formation and breakdown of ABA. Trends in Plant Science 4, 472–478. 10562731 10.1016/s1360-1385(99)01497-1

[CIT0004] Fu DQ Zhu BZ Zhu HL Zhang HX Xie YH Jiang WB Zhao XD Luo KB 2006 Enhancement of virus-induced gene silencing in tomato by low temperature and low humidity. Molecules and Cells 21, 153–160. 16511359

[CIT0005] Fujii H Chinnusamy V Rodrigues A Rubio S Antoni R Park SY Cutler SR Sheen J Rodriguez PL Zhu JK 2009 In vitro reconstitution of an abscisic acid signaling pathway. Nature 462, 660–664. 19924127 10.1038/nature08599 PMC2803041

[CIT0006] Fujimoto SY Ohta M Usui A Shinshi H Ohme-Takagi M 2000 *Arabidopsis* ethylene-responsive element binding factors act as transcriptional activators or repressors of GCC box-mediated gene expression. The Plant Cell 12, 393–404. 10715325 10.1105/tpc.12.3.393 PMC139839

[CIT0007] Giribaldi M Gény L Delrot S Schubert A 2010 Proteomic analysis of the effects of ABA treatments on ripening *Vitis vinifera* berries. Journal of Experimental Botany 61, 2447–2458. 20388747 10.1093/jxb/erq079 PMC2877898

[CIT0008] Hoffmann T Kalinowski G Schwab W 2006 RNAi-induced silencing of gene expression in strawberry fruit (*Fragaria* × *ananassa*) by agroinfiltration: a rapid assay for gene function analysis. The Plant Journal 48, 818–826. 17092319 10.1111/j.1365-313X.2006.02913.x

[CIT0009] Hwang SG Chen HC Huang WY Chu YC Shii CT Cheng WH 2010 Ectopic expression of rice *OsNCED3* in Arabidopsis increases ABA level and alters leaf morphology. Plant Science 178, 12–22.

[CIT0010] Krochko JE Abrams GD Loewen MK Abrams SR Cutler AJ 1998 (+)-Abscisic acid 8′-hydroxylase is a cytochrome P450 monooxygenase. Plant Physiology 118, 849–860. 9808729 10.1104/pp.118.3.849 PMC34795

[CIT0011] Kushiro T Okamoto M Nakabayashi K Yamagishi K Kitamura S Asami T Hirai N Koshiba T Kamiya Y Nambara E 2004 The Arabidopsis cytochrome P450 CYP707A encodes ABA 8′-hydroxylases: key enzymes in ABA catabolism. The EMBO Journal 23, 1647–1656. 15044947 10.1038/sj.emboj.7600121 PMC391058

[CIT0012] Li Q Li P Sun L Wang YP Ji K Sun YF Dai SJ Chen P Duan CR Leng P 2011 Expression analysis of β-glucosidase genes that regulate abscisic acid homeostasis during watermelon (*Citrullus lanatus*) development and under stress conditions. Journal of Plant Physiology 169, 78–85. 21940067 10.1016/j.jplph.2011.08.005

[CIT0013] Li Q Ji K Sun YF Luo H Wang HQ Leng P 2013 The role of *FaBG3* in fruit ripening and *B. cinerea* fungal infection of strawberry. The Plant Journal 76, 24–35. 23802911 10.1111/tpj.12272

[CIT0014] Liu Y Schiff M Dinesh-Kumar SP 2002 Virus-induced gene silencing in tomato. The Plant Journal 31, 777–786. 12220268 10.1046/j.1365-313x.2002.01394.x

[CIT0015] Ma Y Szostkiewicz I Korte A Moes D Yang Y Christmann A Grill E 2009 Regulators of PP2C phosphatase activity function as abscisic acid sensors. Science 324, 1064–1068. 19407143 10.1126/science.1172408

[CIT0016] Manmathan H Shaner D Snelling J Tisserat N Lapitan N 2013 Virus-induced gene silencing of *Arabidopsis thaliana* gene homologues in wheat indentifies genes conferring improved drought tolerance. Journal of Experimental Botany 64, 1381–1392. 23364940 10.1093/jxb/ert003 PMC3598424

[CIT0017] Melcher K Ng LM Zhou XE 2009 A gate-latch-lock mechanism for hormone signaling by abscisic acid receptors. Nature 462, 602–608. 19898420 10.1038/nature08613 PMC2810868

[CIT0018] Nishimura N Hitomi K Arvai AS Rambo RP Hitomi C Cutler SR Schroeder JI Getzoff ED 2009 Structural mechanism of abscisic acid binging and signaling by dimeric PYR1. Science 326, 1373–1379. 19933100 10.1126/science.1181829 PMC2835493

[CIT0019] Ohta M Matsui K Hiratsu K Shinshi H and Ohme-Takagi M 2001 Repression domains of class II ERF transcriptional repressors share an essential motif for active repression. The Plant Cell 13, 1959–1968. 11487705 10.1105/TPC.010127 PMC139139

[CIT0020] Ohta M Ohme-Takagi M Shinshi H 2000 Three ethyleneresponsive transcription factors in tobacco with distinct transactivation functions. The Plant Journal 22, 29–38. 10792818 10.1046/j.1365-313x.2000.00709.x

[CIT0021] Park SY Fung P Nishimura N 2009 Abscisic acid inhibits type 2C protein phosphatases via the PYR/PYL family of START proteins. Science 324, 1068–1071. 19407142 10.1126/science.1173041 PMC2827199

[CIT0022] Qin XQ Zeevaart JAD 2002 Overexpression of a 9-*cis*-epoxycarotenoid dioxygenase gene in *Nicotiana plumbaginifolia* increases abscisic acid and phaseic acid levels and enhances drought tolerance. Plant Physiology 128, 544–551. 11842158 10.1104/pp.010663 PMC148917

[CIT0023] Ren J Chen P Dai SJ Li P Li Q Ji K Wang YP Leng P 2011 Role of abscisic acid and ethylene in sweet cherry fruit maturation: molecular aspects. New Zealand Journal of Crop and Horticultural Science 39, 1–14.

[CIT0024] Ren J Sun L Wu JF Zhao SL Wang CL Wang YP Ji K Leng P 2010 Cloning and expression analysis of cDNAs for ABA 8′-hydroxylase during sweet cherry fruit maturation and under stress conditions. Journal of Plant Physiology 167, 1486–1493. 20728961 10.1016/j.jplph.2010.05.027

[CIT0025] Rodrigo MJ Alquezar B Zacarias L 2006 Cloning and characterization of two 9-*cis*-epoxycarotenoid dioxygenase genes, differentially regulated during fruit maturation and under stress conditions, from orange (*Citrus sinensis* L. Osbeck). Journal of Experimental Botany 57, 633–643. 16396998 10.1093/jxb/erj048

[CIT0026] Saito S Hirai N Matsumoto C Ohigashi H Ohta D Sakata K Mizutani M 2004 Arabidopsis *CYP707A*s encode (+)-abscisic acid 8′-hydroxylase, a key enzyme in the oxidative catabolism of abscisic acid. Plant Physiology 134, 1439–1449. 15064374 10.1104/pp.103.037614 PMC419820

[CIT0027] Santiago J Rodrigues A Saez A Rubio S Antoni R Dupeux F Park SY Marquez JA Cutler SR Rodriguez PL 2009 Modulation of drought resistance by the abscisic acid receptor PYL5 throught inhibition of clade A PP2Cs. The Plant Journal 60, 575–588. 19624469 10.1111/j.1365-313X.2009.03981.x

[CIT0028] Sawada Y Aoki M Nakaminami K 2008 Phytochrome- and gibberellin-mediated regulation of abscisic acid metabolism during germination of photoblastic lettuce seeds. Plant Physiology 146, 1386–1396. 18184730 10.1104/pp.107.115162 PMC2259076

[CIT0029] Schwartz SH Qin XQ Zeevaart JAD 2003 Elucidation of the indirect pathway of abscisic acid biosynthesis by mutants, genes, and enzymes. Plant Physiology 131, 1591–1601. 12692318 10.1104/pp.102.017921 PMC1540303

[CIT0030] Setha S Kondo S Hirai N Ohigashi H 2005 Quantiﬁcation of ABA and its metabolites in sweet cherries using deuterium-labeled internal standards. Plant Growth Regulation 45, 183–188.

[CIT0031] Shang Y Yan L Liu ZQ 2010 The Mg-chelatase H subunit of *Arabidopsis* antagonizes a group of WRKY transcription repressors to relieve ABA-responsive genes of inhibition. The Plant Cell 22, 1909–1935. 20543028 10.1105/tpc.110.073874 PMC2910980

[CIT0032] Sun L Sun Y Zhang M 2012a Suppression of 9-*cis*-epoxycarotenoid dioxygenase, which encodes a key enzyme in abscisic acid biosynthesis, alters fruit texture in transgenic tomato. Plant Physiology 158, 283–298. 22108525 10.1104/pp.111.186866 PMC3252109

[CIT0033] Sun L Wang YP Chen P Ren J Ji K Li Q Li P Dai SJ Leng P 2011 Transcriptional regulation of *SlPYL*, *SlPP2C*, and *SlSnRK2* gene families encoding ABA signal core components during tomato fruit development and drought stress. Journal of Experimental Botany 62, 5659–5669. 21873532 10.1093/jxb/err252 PMC3223059

[CIT0034] Sun L Yuan B Zhang M Wang L Cui MM Wang Q Leng P 2012b Fruit-specific RNAi-mediated suppression of *SlNCED1* increases both lycopene and β-carotene contents in tomato fruit. Journal of Experimental Botany 63, 3097–3108. 22345638 10.1093/jxb/ers026 PMC3350922

[CIT0035] Taylor IB Sonneveld T Bugg TDH Thompson AJ 2005 Regulation and manipulation of the biosynthesis of abscisic acid, including the supply of xanthophyll precursors. Journal of Plant Growth Regulation 24, 253–273.

[CIT0036] Umezawa T Okamoto M Kushiro T Nambara E Oono Y Seki M Kobayashi M Koshiba T Kamiya Y Shinozaki K 2006 CYP707A3, a major ABA 8′-hydroxylase involved in dehydration and rehydration response in *Arabidopsis thaliana* . The Plant Journal 46, 171–182. 16623881 10.1111/j.1365-313X.2006.02683.x

[CIT0037] Wan CY Wilkins TA 1994 A modified hot borate method significantly enhances the yield of high-quality RNA from cotton (*Gossypium hirsutum* L.). Analytical Biochemistry 223, –7–12. 10.1006/abio.1994.1538 7535022

[CIT0038] Wan XR Li L 2006 Regulation of ABA level and water-stress tolerance of *Arabidopsis* by ectopic expression of a peanut 9-*cis*-epoxycarotenoid dioxygenase gene. Biochemical and Biophysical Research Communications 347, 1030–1038. 16870153 10.1016/j.bbrc.2006.07.026

[CIT0039] Wang YP Wang Y Ji K 2013 The role of abscisic acid in regulating cucumber fruit development and ripening and its transcriptional regulation. Plant Physiology and Biochemistry 64, 70–79. 23376370 10.1016/j.plaphy.2012.12.015

[CIT0040] Zhang M Leng P Zhang GL Li XX 2009a Cloning and functional analysis of 9-*cis*-epoxycarotenoid dioxygenase (NCED) genes encoding a key enzyme during abscisic acid biosynthesis from peach and grape fruits. Journal of Plant Physiology 166, 1241–1252. 19307046 10.1016/j.jplph.2009.01.013

[CIT0041] Zhang M Yuan B Leng P 2009b The role of ABA in triggering ethylene biosynthesis and ripening of tomato fruit. Journal of Experimental Botany 60, 1579–1588. 19246595 10.1093/jxb/erp026 PMC2671613

[CIT0042] Zhang YM Yang JF Lu SY Cai JL Guo ZF 2008 Overexpressing *SgNCED1* in tobacco increases ABA level, antioxidant enzyme activities, and stress tolerance. Journal of Plant Growth Regulation 27, 151–158.

