# Supplementary material for: SlNCED1 and SlCYP707A2: key genes involved in ABA metabolism during tomato fruit ripening
Source: J Exp Bot. 2014 Jul 19;65(18):5243–55. doi: 10.1093/jxb/eru288 (PMC4157709; doi:10.1093/jxb/eru288)
Supplement: Supplementary Data [file supp_65_18_5243__index.html]

 SlNCED1 and SlCYP707A2: key genes involved in ABA metabolism during tomato fruit ripening — SlNCED1 and SlCYP707A2: key genes involved in ABA metabolism during tomato fruit ripening — Supplementary Data 

# *SlNCED1* and *SlCYP707A2*: key genes involved in ABA metabolism during tomato fruit ripening

## Supplementary Data

Data files

**Files in this Data Supplement:**

- Supplementary Data - Supplementary Data
