# Supplementary material for: SlNCED1 and SlCYP707A2: key genes involved in ABA metabolism during tomato fruit ripening
Source: J Exp Bot. 2014 Jul 19;65(18):5243–55. doi: 10.1093/jxb/eru288 (PMC4157709; doi:10.1093/jxb/eru288)
Supplement: Supplementary Data [file supp_eru288_jexbot120592_file001.pdf]

***SlNCED1* and *SlCYP707A2*: key genes involved in the ABA metabolism during tomato fruit ripening.** K Ji, W Kai, B Zhao, B Yuan, C Duan, Y Sun, Q Li, S Dai, P Chen, Y Wang, Y Wang, Y Pei, F Xu, and P Leng

SUPPLEMENTARY TABLE S1. Specific primer sequences used for Real-time quantitative PCR

Target gene	Forward primer (5'-3')	Reverse primer (5'-3')
SlSAND	TTGCTTGGAGGAACAGACG	GCAAACAGAACCCCTGAATC
SlNCED1	AGGCAACAGTGAAACTTCCATCAAG	TCCATTAAAGAGGATATTACCGGGGAC
SlNCED2	TGGTTTTCATGGGACATTCATTAGC	ATCTCCCTTCTCAACTCCCTATTCC
SlNCED3	AGGCAACAGTGAAACTTCCATCAAG	CGGTTTGTATAACGGTTGGCGCCG
SlACS2	CTACGCAGCCACTGTCTTTGAC	TGATTCCGACTCTAAATCCTGGTAA
SlACS4	TTGCGACGAAATATATGCTGCT	CACTCGAAATCCTGGAAAACCT
SlACO1	ACTATCCACCATGTCCTAAGCCCG	TCTGTTTGTGCAATTACTCTGTGCAGC
SlETR1	ATGGATGAGAATGGTGTTAGCAGGA	CACAATAAGTGGCCTACCGTGACGT
SlETR3	AAGGGAACCACTGTCACGTTTGTAG	TTAATGTTCTTTGTCACACCAATGTCC
SlETR4	TGAGAATTCGGAAGTTTGGTAGCCA	ACTCCACTCCTATAAGGCACCGTCA
SlETR6	TTGATCATCGGTTTAGCTGCAATTACT	GTCTATTGTAAACGTTACCGTCATGGC
SlCTR1	ACATTTGGATTATGTCAGGCTTGCA	TTGCTCAAACAATGGTTCAAAGAGG
SlCTR3	CTGCAATCATGGATATGCTGAGACCA	CGGAACAAAAGCCTGAAGTAAACAA
SlCTR4	TTATTGAAGCTTGCTGGGTGAATGA	GCCAAATACCAAGTGCAAATGTTTC
SlEIN2	AAGTTCTTGGTGATGTCAGTTCCCC	TCTACTATGCCCTGAAGACGGTTGAG
SlERF2	GAAAAGGGCTCCTCAGAGAGCATAT	TTTCTTCTGCCAATTCAAGTGACGA
SlCYP707A1	CCCAGAGTTCTTTCCTGATCCACAA	GAATGCCACTACCAGATCCTACCAC
SlCYP707A2	TCGAAAAAGGATACAATTCGATGCC	CTGCAATTTGTTCGTCAGTGAGTCC
SlCYP707A3	CTCATGTCCAGGCAATGAGTTAGCC	GGAGCGAAGCTAGAATGAGAATCACC
SlGGPS	TGTCTTGCTGCCTGTGAACTTGTTGG	TACACTTTATGATTCGTCGGCTTTCC
SlPDS	TGGTAGCGAATCAATGGGTCATAAGT	ACCTGCACCAGCAATAACAATCTCC
SlPSY1	GGAGAAGATGCCAGAAGAGGAAGAGT	ACAAGACCAAAGATGCCCATACAGG
SlPSY2	CGTTGTGGCGAAGTATGTGCAGAGTA	TCCAGCCTTGCCTCCCACCTATCTAA
SlZDS	TGCATTGGCATCTCCAGATGATTACT	ATGGGTCTTTACCAGGTCCTTCACG
SlBcyc	GTTCTGAAAGAAGTCATTCGGGTAATG	CATGCCAATAACGAGGTTCTAAGTCA
SlExp	AATCAAATGCGGTTTTAACTGGTCAAT	TCGATTTCTTTTCCTAAGGTGAACAAC
SlXET16	GTGGGATGAACCTGTTATGTCCGAG	TATGATTCGTCTTGTGCTGGTGGTG
